# Caveolin interaction governs Kv1.3 lipid raft targeting

**DOI:** 10.1038/srep22453

**Published:** 2016-03-02

**Authors:** Mireia Pérez-Verdaguer, Jesusa Capera, Ramón Martínez-Mármol, Marta Camps, Núria Comes, Michael M. Tamkun, Antonio Felipe

**Affiliations:** 1 Molecular Physiology Laboratory, Universitat de Barcelona, 08028 Barcelona, Spain; 2 Institut de Biomedicina (IBUB). Departament de Bioquímica i Biologia Molecular. Universitat de Barcelona, 08028 Barcelona, Spain; 3 Clem Jones Centre for Ageing Dementia Research, Queensland Brain Institute, The University of Queensland, Brisbane, Queensland 4072, Australia; 4 Departament Ciències Fisiològiques I, Universitat de Barcelona, 08036 Barcelona, Spain; 5 Department of Biomedical Sciences, Colorado State University, Fort Collins, CO 80523, USA.

## Abstract

The spatial localization of ion channels at the cell surface is crucial for their functional role. Many channels localize in lipid raft microdomains, which are enriched in cholesterol and sphingolipids. Caveolae, specific lipid rafts which concentrate caveolins, harbor signaling molecules and their targets becoming signaling platforms crucial in cell physiology. However, the molecular mechanisms involved in such spatial localization are under debate. Kv1.3 localizes in lipid rafts and participates in the immunological response. We sought to elucidate the mechanisms of Kv1.3 surface targeting, which govern leukocyte physiology. Kv1 channels share a putative caveolin-binding domain located at the intracellular N-terminal of the channel. This motif, lying close to the S1 transmembrane segment, is situated near the T1 tetramerization domain and the determinants involved in the Kvβ subunit association. The highly hydrophobic domain (FQRQVWLLF) interacts with caveolin 1 targeting Kv1.3 to caveolar rafts. However, subtle variations of this cluster, putative ancillary associations and different structural conformations can impair the caveolin recognition, thereby altering channel’s spatial localization. Our results identify a caveolin-binding domain in Kv1 channels and highlight the mechanisms that govern the regulation of channel surface localization during cellular processes.

Subcellular localization of ion channels is essential for proper cell physiology. Lipid raft microdomains, which are enriched with highly packed sphingolipids and cholesterol, have emerged as specific membrane platforms where ion channels converge with signaling molecules, thereby regulating cellular responses^1^. In this context, caveolae, specialized omega-shaped lipid raft microdomains, are abundant in differentiated cell lines such as adipocytes, pneumocytes, endothelial and muscle cells^2^,^3^,^4^. These structures participate in endocytosis, membrane compartmentalization, mechanosensing and mechanoprotection, cell signaling and lipotoxicity protection^5^. Therefore, it is not surprising that an impaired caveolae expression results in extensive physiological dysfunctions^6^. Caveolae are folded by structural proteins called caveolins (Cav), a protein family that is composed of three members of 18–24 KDa (Cav1, Cav2 and Cav3)^7^. Cav participates not only in caveolae biogenesis but also in protein-protein and protein-lipid interactions evoking part of the caveolae properties and functions. The structural associations of Cav during cell signaling are mediated via a Caveolin Scaffolding Domain (CSD) located at the N-terminus of the protein, which interacts with putative Caveolin Binding Domains (CBD) located in target proteins. The CSD of Cav1 and Cav3 recognize a canonical CBD consensus sequence ФxФxxxxФxxФ, with slight variations (where Ф is an aromatic residue and x can be any amino acid)^8^.

K^+^ channels play an essential role in many cellular functions in both excitable and nonexcitable cells. Voltage dependent potassium channels (Kv) participate in controlling repolarization and resting membrane potential. The mammalian Kv1 family (*Shaker*) comprises 8 members (Kv1.1–Kv1.8) which are involved in nerve and muscle physiology^9^. Kv1.3, the third member of the *Shaker* family, is mainly expressed in the nervous and immune systems^10^,^11^ and participates in multiple cellular functions such as the maintenance of the resting membrane potential, immune cell activation, proliferation, cell volume control and apoptosis. Altered Kv1.3 expression is associated with autoimmune diseases, such as multiple sclerosis, rheumatoid arthritis and diabetes^12^, as well as changes in sensory discrimination^13^. In leukocytes, Kv1.3 associates with Kv1.5, another *Shaker* isoform that is crucial for myoblast proliferation^14^,^15^,^16^. Heteromeric channels present specific biophysical and pharmacological properties, as well as different cell surface expressions^14^. Although Kv1.3 and Kv1.5 share lipid raft localization, membrane dynamics suggest distinct membrane microdomain targeting^14^,^16^,^17^,^18^. In this context, differential protein-protein interactions, which influence cell surface expression, have been suggested^17^,^18^. In fact, the Kv1.5-caveolin association is under debate^19^,^20^. In this context, we have previously described the importance of protein interactions influencing Kv1.5 lipid raft targeting and postulate that several partners could compete to determine channel localization^18^.

Here, we study the influence of Cav on the lipid raft targeting of Kv1 channels by analyzing the function of a putative CBD motif conserved in the *Shaker* family. Both Kv1.3 and Kv1.5 target lipid rafts, but only Kv1.3 efficiently interacted with Cav via the CBD where this association is essential for the channel localization in these domains. Moreover, Kv1.3 behavior and activity was conditioned by the presence of Cav1. Therefore, the presence of a CBD near the T1 of Kv1.3 has important functional consequences for Kv1.3 channel physiology.

## Results

### Differential caveolin dependence of Kv1.3 and Kv1.5 lipid raft partitioning

**Figure 1:**
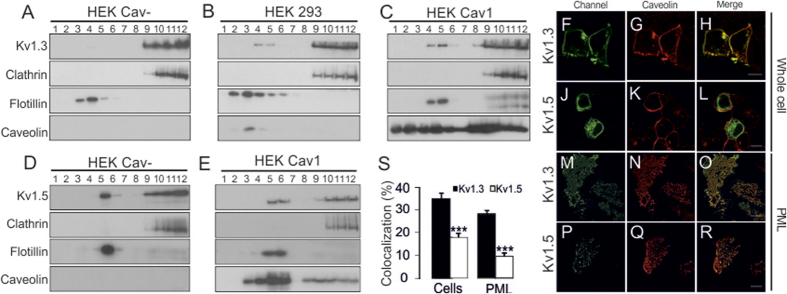
Kv1.3, but not Kv1.5, targeted to lipid raft microdomains in a caveolin-dependent manner. Sucrose-density gradients were performed with total lysates from HEK Cav- (**A**), HEK 293 (**B,D**) and HEK Cav1 (**C,E**) expressing Kv1.3YFP (**A–C**) and Kv1.5YFP (**D,E**). Clathrin and Flotillin were used as non-lipid raft and lipid raft markers, respectively. Note that the overexpression of Cav1 (HEK Cav1) partially targeted caveolin to floating (enriched in lipid raft) and non-floating fractions. Colocalization of Kv1.3 (F_H and **M**–**O**) and Kv1.5 (**J**–**L**,**P**–**R**) with caveolin in whole HEK Cav1 cells (**F–L**) and plasma membrane lawns (PML) (**M**–**R**). Green: channel; Red: caveolin; Merge: colocalization in yellow. Bar scale 10 μm. (**I**) Histogram of the colocalization (%) between channel and caveolin. Closed bars, Kv1.3; open bars, Kv1.5. Data are the mean ± SE of whole-cell (>15 cells) and PML (>35 cells). ***p < 0.001 vs Kv1.3 (Student’s t-test).

Voltage-dependent potassium channels (Kv) from the Kv1 (*Shaker*) family share several structural features that govern channel expression at the cell membrane. To analyze the microdomain targeting of Kv1 channels, three different HEK 293 cell lines exhibiting differential Cav1 expression levels, named HEK Cav-, HEK 293 and HEK Cav1, were used (Supplementary Fig. S1). Thus, Kv1.1-Kv1.5 channels were expressed in HEK Cav- and HEK Cav1 cells. While Kv1.1, Kv1.2 and Kv1.4 showed no relevant lipid raft localization, neither in the presence nor in the absence of Cav1 (Supplementary Fig. S2), Kv1.3 and Kv1.5 partially targeted to raft microdomains (Fig. 1). The steady augmentation of Cav1 increased the targeting of Kv1.3 to floating fractions, which are enriched in lipid raft microdomains and identified by the presence of flotillin (Fig. 1A–C). Unlike Kv1.3, different Cav1 expression levels did not alter Kv1.5 raft association (Fig. 1D,E). As we previously described^17^, while Kv1.3 efficiently targets to the membrane surface (Fig. 1F–H), Kv1.5 was mostly retained intracellularly. In this scenario the presence of Cav1 did not alter this behavior (Fig. 1J–L). In addition, the colocalization between Kv1.3 and Cav1 was higher than that of Kv1.5 (39.5 ± 2.5% and 17.8 ± 1.8%, respectively, p < 0.001, Fig. 1S). To gain further insight into the plasma membrane targeting of Kv1.3 and Kv1.5 and their colocalization with Cav1, we isolated plasma membrane lawns (PML) (Fig. 1M–R). In these preparations, Kv1.3 also showed higher Cav1 colocalization than did Kv1.5 (28.1 ± 1.4% and 9.5 ± 1.4%, respectively, p < 0.001, Fig. 1S). These results suggest that Kv1.3 and Cav1 share spatial localizations at the membrane surface. To understand the extent to which this Kv1.3-Cav1 relationship is physiologically relevant, we analyzed lipid rafts of bone marrow-derived macrophages (BMDM) from Cav1 null mice (Cav1^−/−^). Although Kv1.3 partially localized in low-buoyant fractions in macrophages from both wild-type and Cav1^−/−^ animals, the amount of Kv1.3 in rafts was clearly lower in BMDM from Cav1^−/−^ mice than in those from wt (Supplementary Fig. S3).

### Caveolin 1 associates with Kv1.3 altering channel membrane dynamics, stability and activity

**Figure 2:**
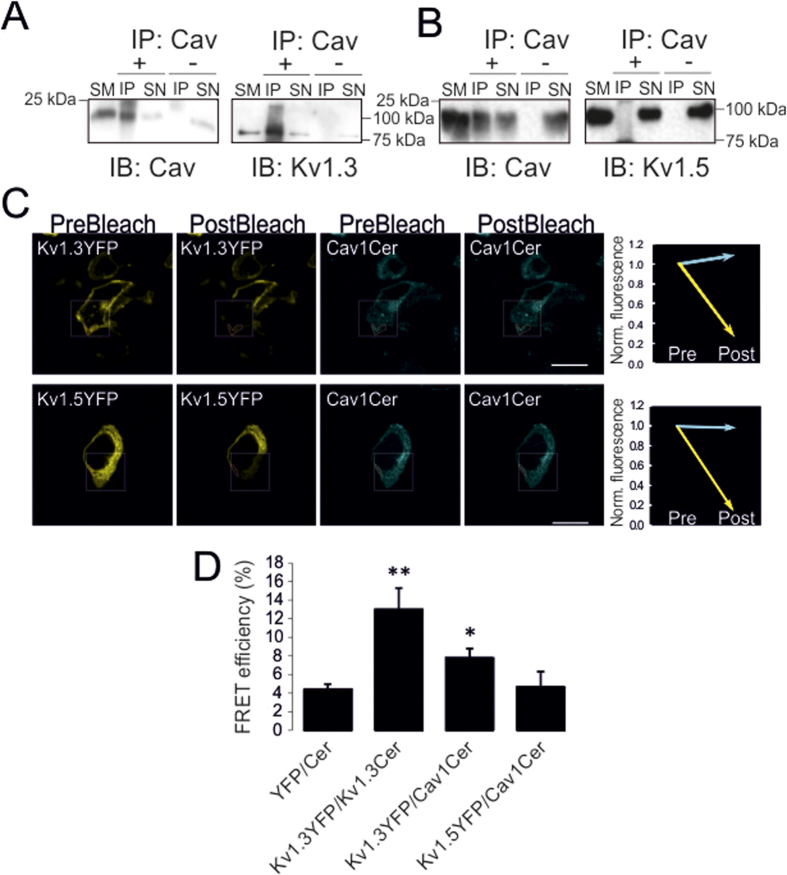
Molecular association of Kv1.3 with caveolin. HEK Cav1 cells were transfected with Kv1.3 (**A**) and Kv1.5 (**B**) and, after lipid raft isolation, floating fractions were subjected to caveolin immunoprecipitation (IP: Cav) in the presence (+) and the absence (−) of caveolin antibody. Blots were analyzed for the presence of Kv1.3 (IB: Kv1.3), Kv1.5 (IB: Kv1.5) and Cav1 (IB: Cav). SM: starting material; SN: Supernatant; IP: immunoprecipitate. (**C**) Representative FRET images of HEK Cav- cotranfected with Cav1-Cerulean and Kv1.3YFP (top panels) or Kv1.5YFP (bottom panels). Images from left to right show acceptor (Channel-YFP) prebleach and postbleach and donor (Cav1-Cerulean) prebleach and postbleach images. Squares insets indicate the bleached zone containing the quantified red limited areas. The line graphs at the right show changes in donor (cyan) and acceptor (yellow) fluorescence after bleach (**D**) Histogram with the FRETeff quantification (%) of YFP-Cer (negative control), Kv1.3YFP-Kv1.3Cer (positive control), and samples from (**C**). Data are the mean ± SE (n > 35). *p < 0.05; **p < 0,01 vs YFP-Cer (Student’s t-test).

The Kv1.3 Cav1-dependent lipid raft localization suggested a putative interaction between Kv1.3 but not Kv1.5 and Cav1. Caveolin interacts with many different signaling proteins such as eNOS (endothelial nitric oxide synthase), Src kinases or PKC, thereby recruiting them into caveolae platforms^21^,^22^,^23^. To directly address this point, we performed coimmunoprecipitation and Förster Resonance Energy Transfer (FRET) experiments. Low-buoyant Cav1 and flotillin-enriched fractions (see Fig. 1) were subjected to caveolin immunoprecipitation. Kv1.3, but not Kv1.5, was coimmunoprecipitated with Cav1 (Fig. 2A,B). Similar results were obtained from clathrin-enriched non-raft fractions (data not shown). Furthermore, FRET results corroborated a molecular interaction between Kv1.3 and Cav1. Thus, when Kv1.3-YFP, but not Kv1.5-YFP, and Cav1-Cer were coexpressed the post-bleching intensity of Cav1-Cer increased (Fig. 2C,D).

**Figure 3:**
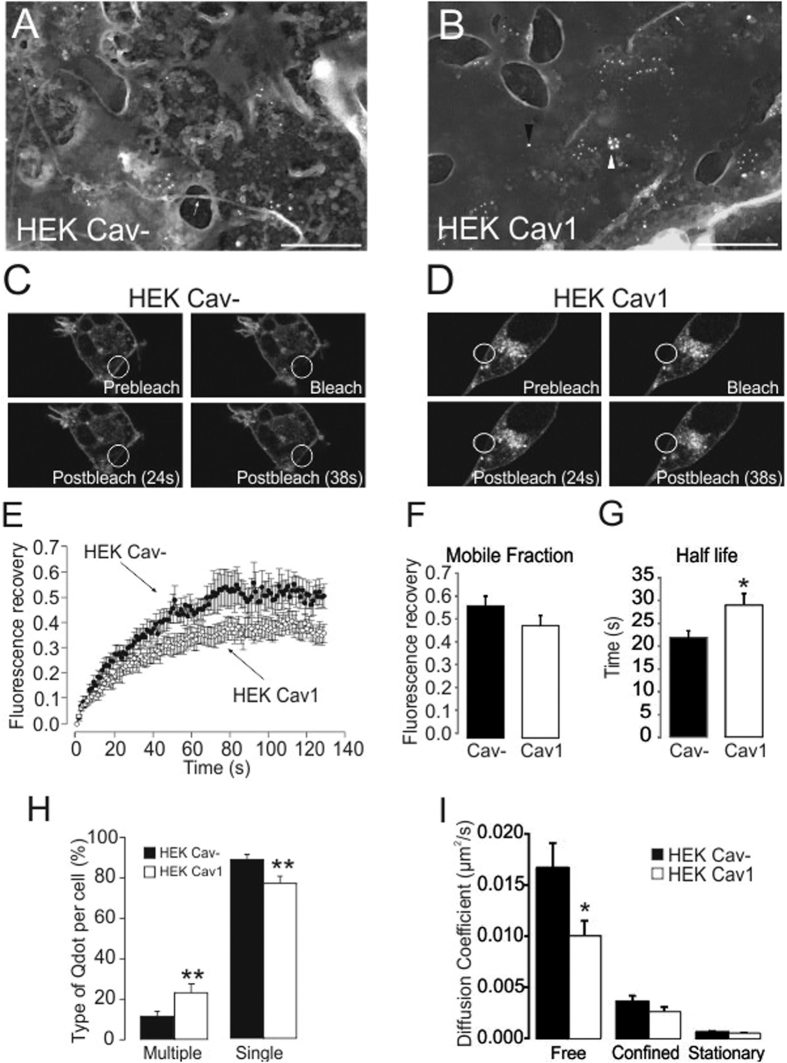
Caveolin expression induces aggregation and slows the lateral diffusion of Kv1.3. (**A,B**) Electronic micrographs of PML from HEK Cav- (**A**) and HEK Cav1 (**B**) expressing Kv1.3. Cav1 and Kv1.3 were immunolocalized with anti-Kv1.3 monoclonal and anti-caveolin polyclonal antibodies and a mixture of 10 nm and 15 nm colloidal gold conjugated secondary antibodies tagging Kv1.3 and caveolin, respectively. Bar Scale 500 nm. White arrows point at microtubules. White arrowhead highlights caveolae. Black arrowhead points at caveolin outside caveolae like structures. (**C–G**) FRAP performed 24 h after Kv1.3YFP transfection in HEK Cav- (**C**) and HEK Cav1 (**D**). Representative images are before (prebleach), during (bleach) and 24 or 38 s after bleach (postbleach). White circles highlight the bleached membrane. (**E**) Fluorescence recovery. (**F**) Mobile fraction and (**G**) half live recovery (the mean ± SE, n = 10). (**H,I**) SPT analysis of HEK Cav- and HEK Cav1 expressing Kv1.3LoopBAD together with BirA for 24 h and tagged for 5 min with Qdots-Streptavidin. (**H**) Qdots were classified as single or multiple units. Histogram displays the relative abundance of both in HEK Cav- and HEK Cav1 (the mean ± SE, n ≥ 15). (**I**) Single Qdots were further classified according to its MSD shape as being free: Brownian movement (linear plot); confined: region restricted movement (decreasing slope plot); or stationary: motionless (diffusion coefficient bellow 0.001 μm^2^/s). Histogram displaying the diffusion coefficient of the different types of single Qdots. Closed bars, HEK Cav-; open bars, HEK Cav1. Data are the mean ± SE (n ≥ 9). *p < 0.05, **p < 0.01 vs HEK Cav- (Student’s t-test).

The caveolin 1 expression induces *de novo* formation of caveolae structures in caveolin-null cells, thereby increasing the plasma membrane structuration^24^,^25^. Moreover, caveolae appear as rigid structures in which caveolins show a reduced mobility^26^. Because Kv1.3 and Cav1 physically interact, the caveolae targeting and the membrane dynamics of Kv1.3 were tested in HEK Cav- and HEK Cav1. Electron micrographs indicated that Kv1.3 was recruited into caveolae-like structures in the presence of Cav1 (Fig. 3A,B). Whether Kv1.3 membrane lateral diffusion was altered in the presence of Cav1 was studied by fluorescence recovery after photobleaching (FRAP) analysis (Fig. 3C–G). The Kv1.3YFP fluorescence recovery was monitored over time in HEK Cav- and HEK Cav1, until a steady state was achieved (Fig. 3E). While the mobile fraction (0.56 ± 0.04 vs 0.48 ± 0.04, for HEK Cav- and HEK Cav1, respectively, n = 10) was similar, the half-life recovery increased in HEK Cav1 (21.77 ± 1.49 s and 28.87 ± 2.49 s for HEK Cav- and HEK Cav1, respectively, p < 0.05, n = 10) (Fig. 3F), where a lower motion of Kv1.3 in the presence of caveolin was observed. This could be explained not only by the major recruitment of the channels in rigid structures, such as caveolae, but also by an increase of liquid-ordered domains structuring the membrane. Therefore, the Kv1.3 membrane dynamics were also analyzed by single particle tracking (SPT) using Qdots (Fig. 3H,I). Single Kv1.3 molecules at the cell surface were tagged with Qdots and monitored by total internal reflection fluorescence (TIRF) imaging with a temporal resolution of 0.5 s (Supplementary video 1). Qdots were classified according to their behavior in single or multiple units (Fig. 3H and Supplementary video 1). The last were defined when more than one Qdot motion agroupated for more than 10 s, thereby suggesting aggregated channels. While the abundance of multiple Qdots increased in HEK Cav1 cells (11.15 ± 2.24% vs 23.84 ± 3.26%, p < 0.05), single Qdots decreased (88.85 ± 2.24% vs 76.15 ± 3.26%, p < 0.01), which suggests an aggregated distribution of Kv1.3 channels in the presence of Cav1. Moreover, the trajectories of single Qdots were analyzed by plotting the mean square displacement (MSD) against time^27^. Three types of motion were observed: (i) simple Brownian diffusion (free), (ii) confined diffusion (confined) and (iii) stationary diffusion (stationary) (Fig. 3I). In this context, the diffusion coefficient of free diffusing channels in the presence of Cav1 decreased (0.016 ± 0.002 vs 0.010 ± 0.001 μm^2^/s for HEK Cav- and HEK Cav1, respectively, p < 0.05). Thus, SPT results suggested both the aggregation of Kv1.3 and reduced channel mobility in the presence of caveolin.

**Figure 4:**
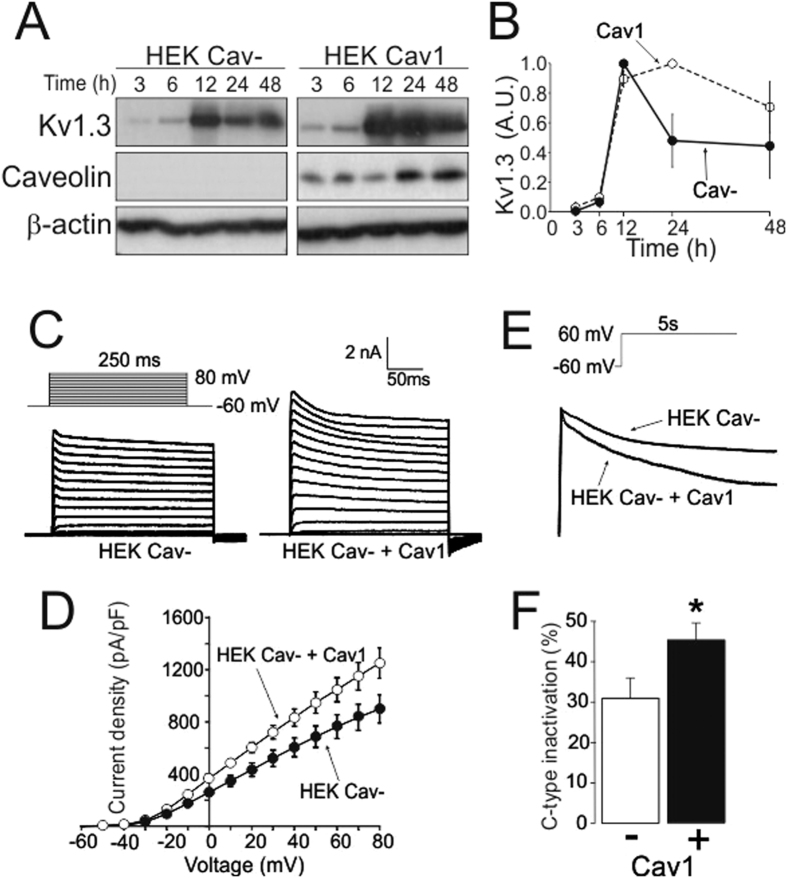
Caveolin 1 stabilized Kv1.3 and modulated current density and inactivation. HEK Cav- and HEK Cav1 cells were transfected with Kv1.3YFP. (**A**) Time course experiments were performed extracting proteins 3, 6, 12, 24 and 48 hours after transfection. (**A**) Representative experiment is shown. Total protein extracts were separated by SDS-PAGE and immunoblotted using Kv1.3, β-actin, and caveolin antibodies. Kv1.3 expression values were corrected by β-actin levels and normalized with the maximum. (●) HEK Cav- cells; (○) HEK Cav1 cells. (**B**) Results are the mean ± SE of two independent experiments. (**C–E**) HEK Cav- cells were transfected with Kv1.3 in the absence (HEK Cav-) or the presence (HEK Cav- +Cav1) of Cav1. (**C**) K^+^ currents were elicited by 250 ms voltage sweeps ranging from −60 mV to +80 mV in 10 mV increments. (**D**) Current density-voltage relationship of HEK Cav- (○) and HEK Cav- +Cav1 (●) cells (mean ± SE of n = 15 and 9 cells, respectively). (**E,F**) C-type inactivation. (**E**) Representative traces evoked by a 5 s pulse from −60 mV to +60 mV. (**F**) Remaining current density (%) at the end of the pulse shown as the mean ± SE of six cells both in the HEK Cav- (Cav1 -) and the HEK Cav- +Cav1 (Cav1+). *p < 0.05 vs Cav- (Student’s t-test).

**Figure 5:**
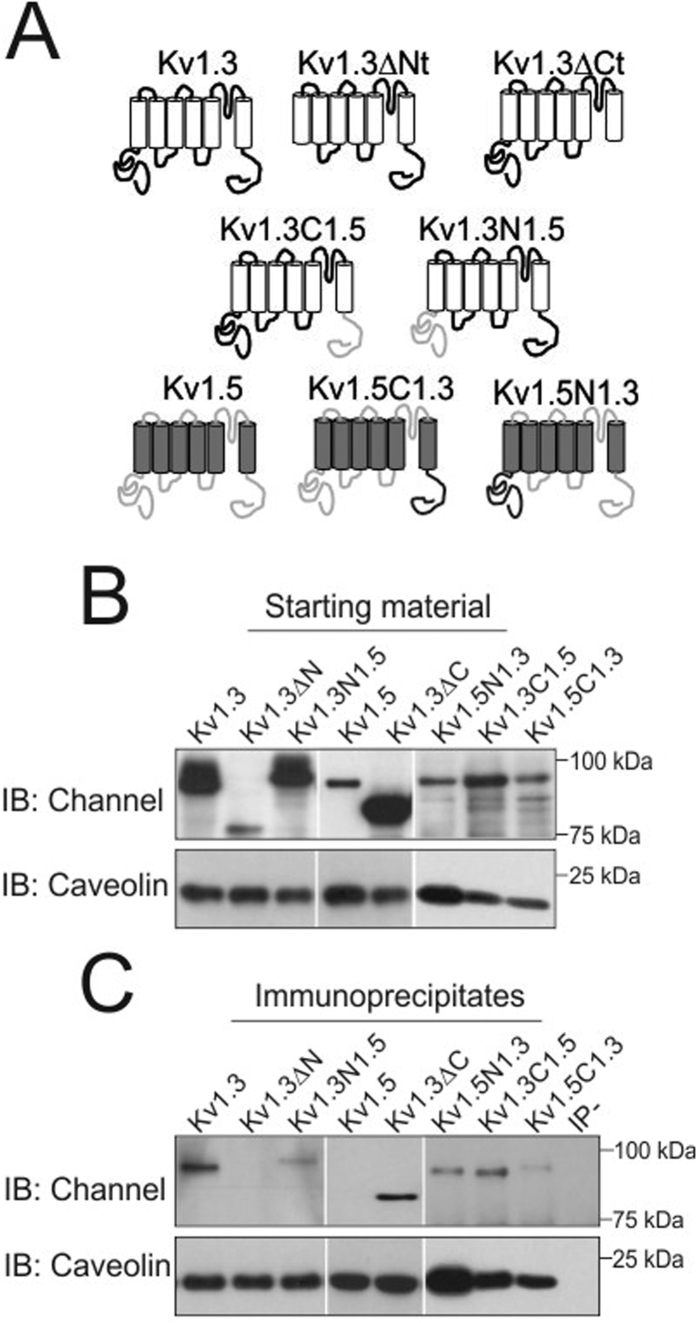
The N-terminal domain of Kv1.3 is essential for the interaction with caveolin. Total protein lysates of HEK Cav1 obtained 24 h after transfection with the indicated channel (tagged with YFP) were subjected to immunoprecipitation against caveolin. Samples separated by SDS-PAGE and immunoblotted (IB) against GFP (channel) and caveolin antibodies. (**A**) Schematic diagrams of Kv1.3 truncated channels and Kv1.3-Kv1.5 chimeras. Kv1.3 domains: white barrels and black lines. Kv1.5 domains: gray barrels and gray lines. (**B**) Starting materials. Top panel, filters were immunobloted against GFP (Channels). Bottom panel, filters were probed against Cav to demonstrate Cav immunoprecipitation. (**C**) Immunoprecipitates. No immunoprecipitation was observed in the absence of antibody (IP-).

Glucose transporter type 4 (Glut4) and insulin receptor (IR) are recruited in caveolae^28^, and Cav1 participates in Glut4 and IR stability. Thus, Cav1 depletion reduces Glut4 and IR protein abundance by their faster degradation^29^. Therefore, we next studied whether Cav1 association altered Kv1.3 stability. Time-course experiments performed in Cav- and Cav1 HEK cells demonstrated that, similar to Glut4 and IR, Kv1.3 persisted for a longer period of time in the presence of Cav1 (Fig. 4A,B). In this context, Cav1 can also affect channel activity^1^. Therefore, for these experiments, Cav1 was reintroduced into HEK Cav- cells, and Kv1.3 electrophysiological properties were analyzed. While the threshold of activation was similar, the presence of Cav1 increased the Kv1.3 current density (Fig. 4C,D). Slow C-type inactivation is a characteristic of Kv1.3. It involves conformational changes of the channel that result in closure of the external mouth of the pore with probable cooperativity between subunits^30^. In this context, the Cav1 interaction enhanced the C-type inactivation of Kv1.3. Thus, the current at the end of a 5 s pulse (+60 mV) was lower in the presence than in the absence of Cav1 (Fig. 5E,F).

### Caveolin 1 interacts with Kv1.3 via a CBD signature located at the N-terminal of the channel

**Figure 6:**
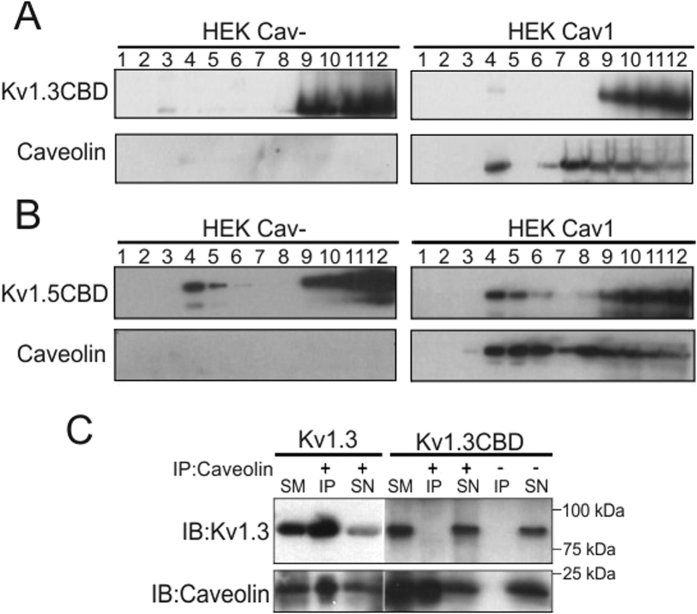
The CBD at the N-terminal domain is responsible for the targeting of Kv1.3 to lipid raft and the association with Cav1. Lipid raft isolations performed in HEK Cav- (left panels) and HEK Cav1 (right panels) cells expressing Kv1.3CBD (**A**) and the Kv1.5CBD (**B**) mutant channels (^166^FQRQVWLLF^174^ to AQRQVGLLA and ^232^FQRQVWLIF^240^ to AQRQVGLIA, respectively). (**C**) HEK Cav1 expressing either Kv1.3 or Kv1.3CBD were subjected to immunoprecipitation against caveolin (IP: Cav1) in the presence (+) and the absence (−) of caveolin antibody. Samples were separated by SDS-PAGE and immunoblotted against Kv1.3 (IB:Kv1.3) and caveolin (IB:Caveolin) SM: starting material; SN: Supernatant; IP: immunoprecipitate.

The N-terminus of Kv1 channels contains important signatures involving tetramerization and regulatory subunit association. Although caveolin uses a CSD to interact with substrates via a CBD^7^,^8^,^31^, this model has been compromised by structural and bioinformatic analysis^32^. In this context, the N-terminus of Kv1.1-Kv1.5 contains putative CBDs lying next to the first transmembrane segment (amino acids 166 to 174 in rKv1.3), right after the T1 tetramerization domain and the Kvβ subunit association signature (Supplementary Fig. S2G). This CBD is represented by a ФxxxxФxxФ consensus sequence, where Ф is a hydrophobic residue. Our data indicates that Kv1.3 and Kv1.5 localized significantly in rafts but only Kv1.3 directly interacted with Cav1. Therefore, we next focused on whether the Kv1.3 CBD molecular determinant was involved in Cav1 interaction. To do so, we performed coimmunoprecipitation assays with different Kv1.3 mutants and Kv1.3-Kv1.5 chimerical channels (Figs 5 and 6). While Kv1.3ΔCt (no C-terminal domain) coimmunoprecipitated with Cav1, Kv1.3ΔNt (no N-terminal domain) did not. In this scenario, to rule out the effect of an altered tetramer formation on traffic and subcellular localization of the Kv1.3 truncated channels, we analyzed Kv1.3/Kv1.5 chimeras that preserved the full integrity of the channel. Chimeras, containing the Kv1.3 N-terminal domain coimmunoprecipitated with Cav1 to a greater extent than did chimers that contained the N-terminus of Kv1.5 (Fig. 5B,C). To further understand this specific Kv1.3 signature, the putative CBD of Kv1.3 and Kv1.5 was mutated. Thus, aromatic amino acids were substituted by alanine or glycine-generating CBD mutants (Kv1.3: ^166^FQRQVWLLF^174^ to ^166^AQRQVGLLA^174^; Kv1.5: ^232^FQRQVWLIF^240^ to ^232^AQRQVGLIA^240^). While the Kv1.3 mutant (Kv1.3CBD) showed a reduced lipid raft partitioning with no Cav1 dependency (Fig. 6A), the Kv1.5CBD showed no lipid raft targeting alterations (Fig. 6B). Moreover, the Kv1.3CBD did not coimmunoprecipitate with Cav1, thereby highlighting the importance of the CBD integrity for Kv1.3 interactions with Cav1 (Fig. 6C).

We found no interactions between Kv1.5 and Cav1; however, by converting the putative Kv1.5 CBD to that of Kv1.3 (Kv1.5I239L), we observed a positive Cav1 coimmunoprecipitation Fig. S4). Furthermore, evidence suggests that Kv1.5 could interact indirectly with caveolins by the formation of supramolecular complexes, including SAP97^33^,^34^. SAP97 (synapse-associated protein 97) is a member of the membrane associated guanylate kinase (MAGUK) family that also includes PSD95 (postsynaptic density protein 95). Therefore, we expressed Kv1.5 in the presence and absence of PSD95 in HEK Cav1 cells. In this scenario, Kv1.5 coimmunoprecipitated with Cav1, only in the presence of PSD95 (Fig. S4). Therefore, our data suggest that, while the full integrity of the Kv1.3 CBD is sufficient to interact with Cav1, Kv1.5 requires ancillary proteins.

## Discussion

Evidence demonstrates that Kv1.3 targets specific membrane localizations^35^,^36^, and location displacements entail pathological consequences^37^. Our study highlights the main mechanism of Kv1.3 channel membrane surface partitioning. We report here that, among Kv1 (*Shaker*) channels, only Kv1.3 and Kv1.5 targeted significantly to lipid rafts. However, while the Kv1.5 floatability was independent of the caveolin expression, Kv1.3 lipid raft targeting increased in a caveolin dose-dependent manner. This is of physiological relevance because this was confirmed in BMDM from Cav1^−/−^ mice. Furthermore, caveolin-channel colocalization was higher with Kv1.3 than with Kv1.5. Finally, we have clearly identified a CBD that is located at the N-terminal domain of Kv1.3 and is the responsible element for Cav1 interaction and lipid raft localization of the channel. Because lipid raft targeting has been proposed as a mechanism for ion channel regulation^1^,^38^, our results contribute to this expanding field.

Kv1.3 redistribution within the plasma membrane is critical for lymphocyte physiology^37^. Upon activation, T cells spatially reorganize membrane proteins to form the immunological synapse (IS), where lipid rafts accumulate and recruit TCR (T-cell receptor), CD3 (cluster of differentiation protein 3) and Kv1.3^10^. Caveolin is crucial for the IS reorganization of CD8 T cells^39^. Thus, the Kv1.3-caveolin interaction described here could participate in the recruitment of Kv1.3 into the IS orchestrated by caveolin. Moreover, the cell membrane composition and lipid raft integrity regulates Kv1.3 activity^40^. This is in agreement with the functional consequences that are observed when Kv1.3 rearranges into the IS^41^. Similarly, Kv1.3 activity was altered in the presence of caveolin. Caveolins also regulate the activity of other channels such as Nav1.5^42^. In addition, caveolin 3 also coimmunoprecipitates with cardiac Kv11.1^43^,^44^; however, a direct interaction between caveolins and channels is not a unique way to target channels to raft domains. In this context, our Kv1.5 data are in the same line of evidence as that described for Kv1.4. The location of Kv1.4 in caveolar domains is uncertain, but the presence of PSD95 increases the targeting to rafts microdomains^45^. Conversely, the raft localization of Kv2.1 and Kv4.2 seems independent of the presence of auxiliary scaffolding proteins such as caveolins or PDZ-containing proteins^45^,^46^. In this scenario, much work must be conducted to decipher different partnership associations, thereby conforming specific cell channelosomes, which allows for the spatial localization of channels and the regulation of physiological response.

Evidence suggests an increasing number of ion channels, mostly cardiac, are in caveolar rafts^47^. However, our results confirmed, for the first time, that Kv1.3 lipid raft targeting occurs via a direct interaction wherein caveolin recruits the channel inside caveolae structures, thereby restricting the channel’s lateral diffusion. The molecular determinant of Kv1.3 that is involved in such interaction is a CBD located at the N-terminus of the channel in close proximity to the T1 tetramerization domain and the Kvβ subunit interaction signature. Although other Kv1 members share similar motifs, none displayed a caveolin-dependent behavior or major lipid raft targeting. Our results demonstrated that few, single point differences within the CBD signature and/or impaired CBD accessibility due to bulky intracellular domains, may impair this interaction. Interestingly, coimmunoprecipitation studies using chimeric Kv1.3/Kv1.5 and Kv1.5(I239L) mutant channels suggest both. Thus, while Kv1.5Nt, containing a CBD motif, was not enough for Kv1.5-Cav1 coimmunoprecipitation, a fairly positive association was observed when the bulky C-terminal of Kv1.5 was substituted by the C-terminal of Kv1.3. In addition, the introduction of a di-leucine motif, within the CBD of Kv1.5 (Kv1.5 I239L), triggered Cav1 co-immunoprecipitation. Our data suggest that the balance of other interacting motifs within Kv1.5 could mask the CBD accessibility and/or effectiveness^18^, which is similar to what has been previously reported for other forward trafficking signals, such as VxxSL or YMVIEE^48^,^49^. In this sense, Kv1.2, containing the same CBD of Kv1.3, lacks strong trafficking signals and exhibits endoplasmic reticulum retention^50^. Unlike Kv1.3 that colocalizes with caveolin in Golgi^17^, Kv1.2 and caveolin do not share intracellular compartments what would impair the association. However, Kv1.2 cell surface is promoted by PSD95 and Kvβ subunits^51^. It is tempting to speculate that different structural tertiary configurations of bulky domains could condition protein–protein interactions with MAGUK proteins, such as PSD95 or SAP97, that interact differently with Kv channels^37^,^38^,^39^. This could be explained by supramolecular complexes formed by Kv1.5, Cav1 and SAP97, which further supports our Kv1.5 and PSD95 data in HEK Cav1 cells^33^,^52^,^53^.

In summary, our results help to elucidate the mechanisms that target Kv1 channels to specific surface microdomains that participate in fine-tuning the cellular responses. Unlike other neuronal Kv1 channels, Kv1.3 interacts with caveolin through a CBD placed at the N-terminal domain of the channel adjacent to the first transmembrane segment and in close proximity to the T1 domain and the Kvβ binding site. This association targets Kv1.3 to caveolar structures that regulate both the channel membrane dynamics and activity.

## Methods

### Expression plasmids and site-directed mutagenesis

Rat Kv1.3 in pRcCMV was provided by T.C. Holmes (University of California, Irvine, CA). Rat Kv1.1 and Kv1.4 in pGEM7 and human Kv1.5 in pBK constructs were subcloned into pEYFP-C1 and pECerulean-C1 (Clontech). Kv1.3/Kv1.5 chimeras were generated in the pEYFP-rKv1.3 and pEYFP-hKv1.5 channels by inserting BglII and EcoRI sites in the N- and C-terminal domains of the channels. Mutants were generated using the QuikChange site-directed mutagenesis kits (Stratagene). LoopBAD (BAD, Biotin acceptor domain) sequence was inserted in the first extracellular loop of pEYFP-Kv1.3 within a preexisting NruI site for rKv1.3LoopBAD construct. *E. coli* Biotin ligase containing construct pBtac_BirA was used as previously described^54^. Rat Cav 1 into pECerulean-C1 was provided from J.R. Martens (University of Florida Medical School). Cav1 was inserted into pcDNA3 by digestion of Cav-pECerulean (HindIII-BamHI). Constructs were verified by sequencing.

### Cell culture, transient transfections and raft isolation

HEK 293 cells were grown in DMEM containing 10% FBS and 100 U/ml penicillin/streptomycin (Gibco). Transient transfection was performed using MetafecteneTM Pro (Biontex) at nearly 80% confluence. Murine bone marrow derived macrophages were isolated, as previously described^55^. All of the experiments and surgical protocols were performed in accordance with the guidelines approved by the ethical committee of the Universitat de Barcelona following the European Community Council Directive 86/609 EEC.

Low density, Triton-insoluble complexes were isolated, as previously described^18^,^20^. Cells were homogenized in 1 ml of 1% Triton X-100, and sucrose was added to a final concentration of 40%. A 5–30% linear sucrose gradient was layered on top and further centrifuged (39,000 rpm) for 20–22 h at 4 °C in a Beckman SW41 rotor. Gradient fractions (1 ml) were collected from the top and analyzed by Western blot.

### Protein extraction, co-immunoprecipitation and western blot analysis

Cells, washed in cold PBS, were lysed on ice with NHG solution (1% Triton X-100, 10% glycerol, 50 mmol/L HEPES pH 7.2, 150 mmol/L NaCl) supplemented with 1 μg/ml of aprotinin, 1 μg/ml of leupeptin, 1 μg/ml of pepstatin and 1 mM of phenylmethylsulfonyl fluoride to inhibit proteases. Homogenates were centrifuged at 16,000 × g for 15 min, and the protein content was measured using the Bio-Rad Protein Assay.

For immunoprecipitation, samples were precleared with 30 μl of protein A-Sepharose beads for 2 h at 4 °C with gentle mixing as part of the co-immunoprecipitation procedures. The beads were then removed by centrifugation at 1,000 × g for 30 s at 4 °C. Samples were incubated overnight with the anti-caveolin antibody (4 ng/μg protein) at 4 °C with gentle agitation. Thirty microliters of protein A-Sepharose were added to each sample and incubated for 4 h at 4 °C. The beads were removed by centrifugation at 1,000 × g for 30 s at 4 °C, washed four times in NHG, and resuspended in 100 μl of Laemmli SDS buffer.

Protein samples (50 μg), raft fractions (50 μl) and immunoprecipitates were boiled in Laemmli SDS loading buffer and separated by 10% SDS-PAGE. Next, samples were transferred to PVDF membranes (Immobilon-P, Millipore) and blocked with 5% dry milk-supplemented 0.05% Tween 20 PBS. The filters were then immunoblotted with specific antibodies: anti-GFP (1/1,000, Roche), anti-caveolin (1/2,500, BD Biosciences), anti-Kv1.3 (1/500, Neuromab), anti-clathrin (1/1,000, BD Biosciences), anti-flotillin (1/1,000, BD Biosciences). Finally, filters were washed with 0.05% Tween 20 PBS and incubated with horseradish peroxidase conjugated secondary antibodies (BioRad).

### Immunocytochemistry, plasma membrane lawns (PML) and transmission electron microscopy

HEK cells seeded on poly-D-lysine-treated coverslips were used 24 h after transfection (Metafectene Pro). Cells were washed in PBS (phosphate-buffered saline without K^+^) and fixed with 4% paraformaldehyde for 10 min at room temperature (RT). To detect Cav 1, cells were permeabilized using 0.1% Triton X-100 for 10 min. After a 60 min in blocking solution (10% goat serum (Gibco), 5% non-fat dry milk, PBS), cells were treated with rabbit anti-caveolin (1/100, BD Biosciences) antibody in 10% goat serum, 0.05% Triton X-100 and again incubated for 1 h. After 3 washes, preparations were incubated for 45 min with Alexa-Fluor-555 conjugated antibody (1:500; Molecular Probes), washed and mounted in Mowiol (Calbiochem). All procedures were performed at RT.

PML preparations were obtained via osmotic shock with minor modifications^29^. Briefly, cells were cooled on ice for 5 min and washed twice in PBS. Next, cells were incubated for 5 min in 1/3 KHMgE (70 mM KCl, 30 mM HEPES, 5 mM MgCl2, 3 mM EGTA, pH 7.5) and gently washed with non-diluted KHMgE to induce the hypotonic shock. Busted cells were removed from the coverslip by pipetting up and down. After two washes with KHMgE buffer only membrane sheets remained attached. PML were fixed with fresh 4% paraformaldehyde for 10 min at room temperature and mounted in Mowiol mounting media.

For transmission electron microscopy, PML were treated as performed for immunocytochemistry, but visualized with different secondary antibodies. Kv1.3 was recognized by a mouse anti-Kv1.3 antibody (1/20, Neuromab). Goat anti-mouse and anti-rabbit secondary antibodies, conjugated to 10 nm and 15 nm gold particles, recognized Kv1.3 and Cav1, respectively. Briefly, processed samples were further fixed with 2.5% glutaraldehyde in PBS for 30 min at RT. Next, samples were subjected to freeze-drying, washed and cryoprotected with 10% methanol. Samples were then cryofixed using slam-freezing (BAF-060, Bal-Tec) for 90 min at −90 °C and 10–7 mbar pressure. Replicas were obtained by rotationally (136 rpm) evaporating 1 nm platinum through electron cannon (at an angle of 23°). This was reinforced by evaporating 10 nm carbon (at an angle of 75°). Replicas were separated from the sample using 30% fluorhydric acid. Finally, samples were washed and mounted over Formvar coated grilles.

### Föster resonance energy transfer (FRET)

FRET was performed in the acceptor photobleaching configuration. Samples were imaged with a Leica SP2 confocal microscope. Images were acquired before and after YFP bleach using 63 × oil immersion objective at zoom 4. Excitation was via the 458 and 514 nm lines of the Ar laser, and 473–495 and 535–583 bandpass emission filters were used. FRET efficiency (FRETeff) was calculated using the equation:


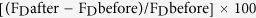


where, F_D_after: donor fluorescence (Cerulean) after and F_D_before before acceptor (YFP) bleach. Analysis was performed using ImageJ.

### Fluorescence recovery after photobleaching (FRAP) and Single particle tracking (SPT)

Experiments were performed as previously described^46^,^54^,^56^. For FRAP experiments, an Olympus FV1000 microscope was used. Briefly, YFP was bleached during 250 ms with a 515 nm line Ar laser at 30% and was fluorescence monitored before and after bleach with a PLAPO 60x NA 1:1,40 oil objective at zoom 4 acquiring every 1.108 s. Acquisition was performed with the 515 nm Ar laser line at 1% and a 525–560 nm bandpass emission filter.

SPT analysis was performed as previously described^46^. Briefly, cells co-expressing Kv1.3LoopBAD and BirA for 24 h were incubated for 5 min at RT with 0.1 nM Streptavidin Qdots655 (Invitrogen, Oregon) in 1% BSA HIS (146 mM NaCl, 4.7 mM KCl, 2.5 mM CaCl_2_ × 2H_2_O, 0.6 mM MgSO_4_ × 7H_2_O, 0.15 mM NaH_2_PO_4_ × 2H_2_O, 0.1 mM ascorbic acid, 8 mM Glucose, 20 mM HEPES, pH 7.4) and washed five times with HIS at RT. Cells were imaged in the following hour at 37 °C in a 5% CO_2_ atmosphere. Imaging was performed with Nikon Eclipse Ti PerfectFocus equipped TIRF (Total internal reflection fluorescence) microscope with a 100xPlanApoTIRF, 1.49 NA, oil objective. YFP was excited with 488 nm line Ar laser at 2% and Qdots with 561 nm laser at 20%. Emission was collected through a Sutter Lambda 10–3 filter wheel and recorded with an Andor iXon EMCCD DUD897 camera. For TIRF acquisition, the incident angle was 63.3°. Imaging acquisition was approximately 10 MHz. Videos were processed using Volocity (PerkinElmer Software). SPT was performed manually. The tracks were then analyzed using Sigmaplot to obtain mean square displacement (MSD) and the Diffusion Coefficient.

### Electrophysiology

Patch–clamp whole-cell configuration experiments were performed, as performed in^49^. To evoke voltage-gated currents, cells were stimulated with 250 ms square pulses ranging from −60 to +80 mV in 10 mV steps. C-type inactivation was studied by applying a long pulse of 5 s at +60 mV. The peak amplitude (pA) was normalized using the capacitance values (pF). Data analysis was performed using FitMaster (HEKA) and Sigma Plot 10.0 software (Systat Software). All recordings were performed at RT.

## Additional Information

**How to cite this article**: Pérez-Verdaguer, M. *et al.* Caveolin interaction governs Kv1.3 lipid raft targeting. *Sci. Rep.* **6**, 22453; doi: 10.1038/srep22453 (2016).

## Supplementary Material

Supplementary Information

Supplementary Video 1

Supplementary Video 2
