# Supplementary material for: Caveolin interaction governs Kv1.3 lipid raft targeting
Source: Sci Rep. 2016 Mar 2;6:22453. doi: 10.1038/srep22453 (PMC4773814; doi:10.1038/srep22453)
Supplement: Supplementary Information [file srep22453-s1.pdf]

Supplementary Information

Caveolin interaction governs Kv1.3 lipid raft targeting

Mireia Pérez-Verdaguer, Jesusa Capera, Ramón Martínez-Mármol, Marta Camps, Núria Comes, Michael M. Tamkun and Antonio Felipe

Legend to Supplementary Figures

Supplementary Figure 1. Caveolin expression in different HEK 293 cell lines. Representative western blot of total protein lysates from HEK 293 cells (regular HEK cell line), HEK Cav1 (stably overexpressing caveolin 1) and HEK Cav- (lentiviral ablation of Cav1) and. β-actin was used as a loading control.

Supplementary Figure 2. Lipid raft distribution of Kv1 channels. rKv1.1, rKv1.2 and rKv1.4 YFP-tagged channels were transfected in HEK Cav- (left panels) and HEK Cav1 (right panels). Lipid rafts were isolated using a sucrose gradient and the channel distribution was analyzed by western blot against GFP. (A, B) Kv1.1; (C, D) Kv1.2; (E, F) Kv1.4. While caveolin was used as a raft marker, clathrin indicated non-raft fractions. Flotillin highlighted raft fractions, despite the absence of caveolin in HEK Cav- cells. (G) Putative CBD sequence in the N-terminal domain of Kv1.1-Kv1.5 channels. Identities appear highlighted in gray. * denotes hydrophobic residues of the putative ΦXXXXΦXXΦ motif.

Supplementary Figure 3. Lipid raft distribution of Kv1.3 in macrophages. (A-B) Murine bone marrow derived macrophages (BMDM) were isolated from wild-type C57BL/6 (BMDM $Cav1^{+/+}$) and Cav null (BMDM $Cav1^{-/-}$) mice. Total lysates from BMDM were subjected to lipid raft isolation. Note that while flotillin was situated in low-buoyant density fractions in both cases, Cav1 was only expressed in wild type animals.

Supplementary Figure 4. The Kv1.5 (I239L) mutant and the presence of PSD95 trigger the co-immunoprecipitation of Kv1.5 and caveolin. Total protein lysates of HEK Cav1 were obtained 24 h after transfection with Kv1.5 wt, in the presence or the absence of PSD95, and Kv1.5(I239L). See Supplementary Fig. S2G for details. Samples were immunoprecipitated against caveolin (IP: Cav) and immunoblotted against Kv1.5 (IB:Kv1.5) and caveolin (IB: Cav1) antibodies. Left panels, Starting materials (SM). Right panels, Immunoprecipitates (IP). IP- negative control in the absence of the anti-Cav antibody.

Legend to Supplementary Videos

Supplementary video 1. Representative TIRF video of HEK Cav- cells 24 h after cotransfection with Kv1.3LoopBAD and BirA. Cells were tagged for 5 min with Qdots. Qdots were classified as single or multiple units (see Fig. 4). Several Qdots with independent motions were classified as single, while only a few moved together in small groups and were classified as multiple.

Supplementary video 2. Representative TIRF video of HEK Cav1 cells 24 h after cotransfection with Kv1.3LoopBAD and BirA. Cells were tagged for 5 min with Qdots. Qdots were classified as single or multiple units (see Fig. 4). Several Qdots with independent motions were classified as single, while only a few moved together in small groups and were classified as multiple. White arrows point at some multiple Qdots.

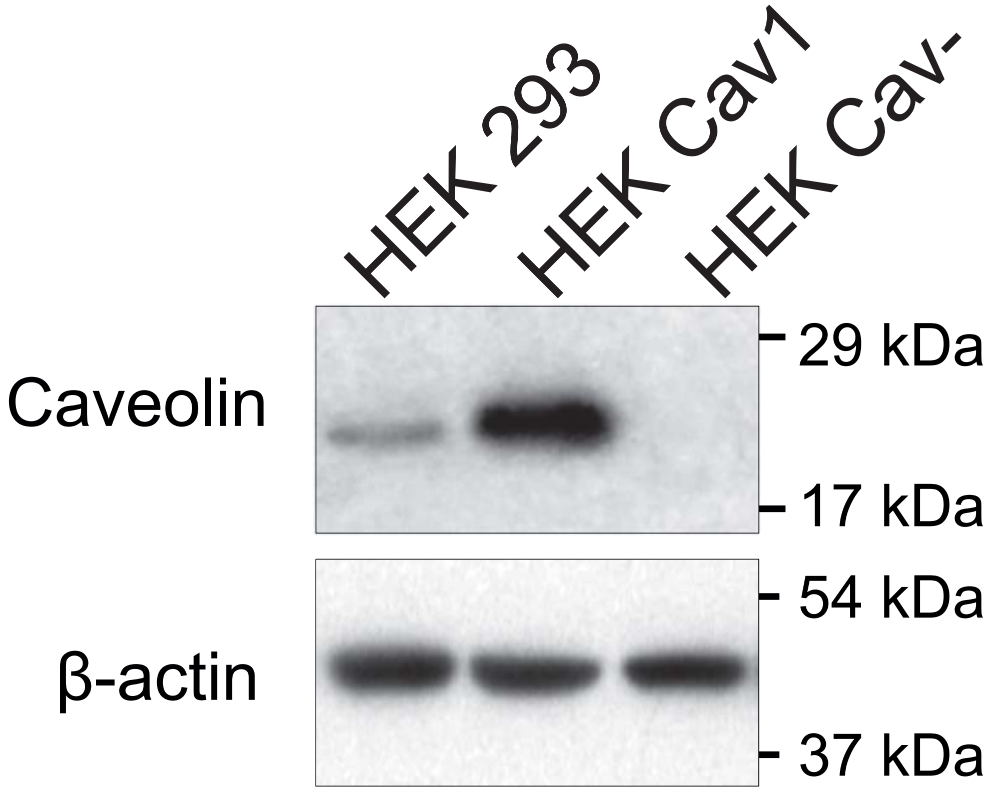
HEK 293
HEK Cav1
HEK Cav-
Caveolin
29 kDa
17 kDa
β-actin
54 kDa
37 kDa

Figure S1

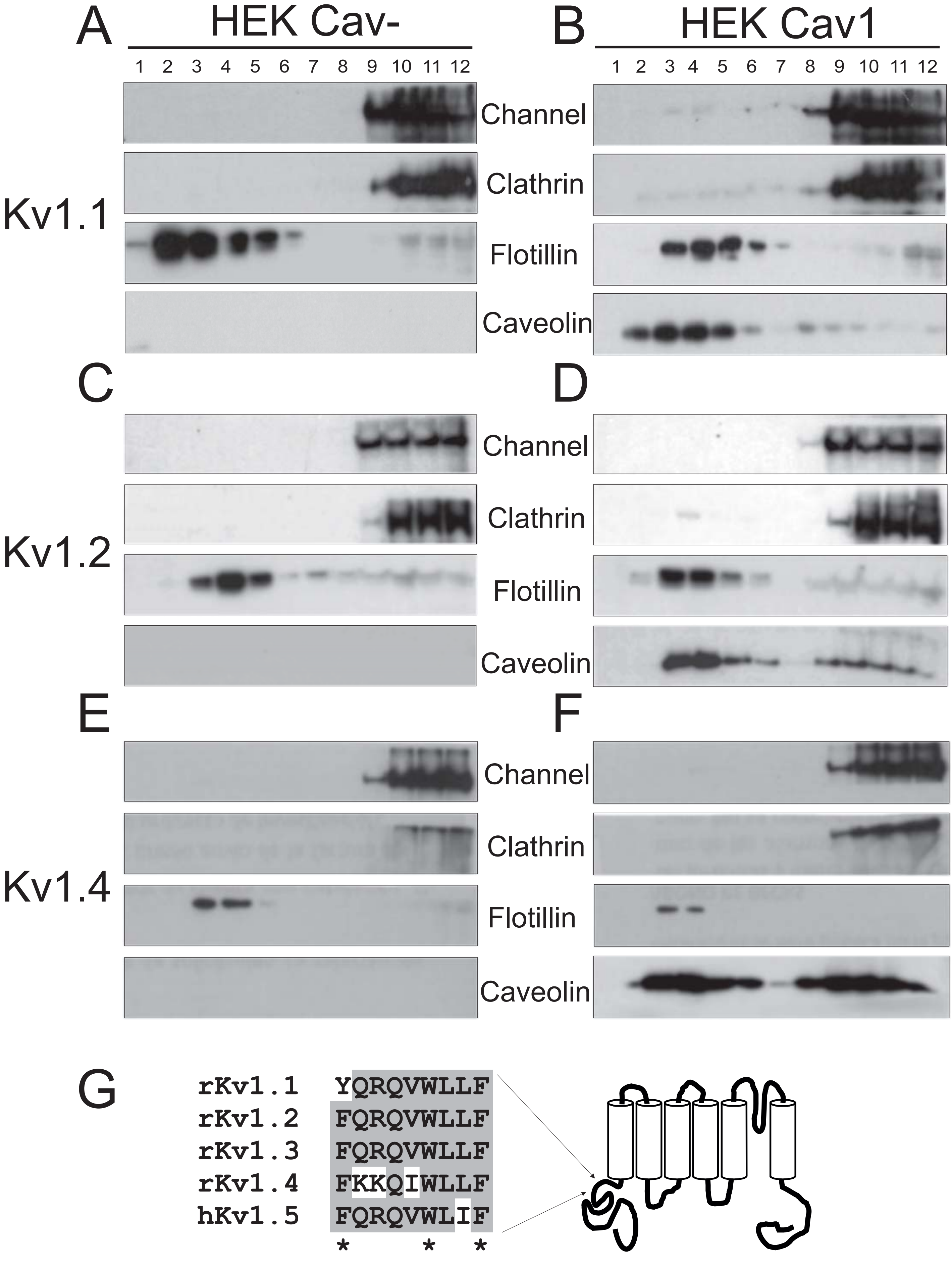

A
HEK Cav-
B
HEK Cav1
1 2 3 4 5 6 7 8 9 10 11 12
1 2 3 4 5 6 7 8 9 10 11 12
Channel
Clathrin
Kv1.1
Flotillin
Caveolin
C
D
Channel
Clathrin
Kv1.2
Flotillin
Caveolin
E
F
Channel
Clathrin
Kv1.4
Flotillin
Caveolin
G
rKv1.1 YQRQVWLLF
rKv1.2 FQRQVWLLF
rKv1.3 FQRQVWLLF
rKv1.4 FKKQIWLLF
hKv1.5 FQRQVWLIF
* * *

Figure S2

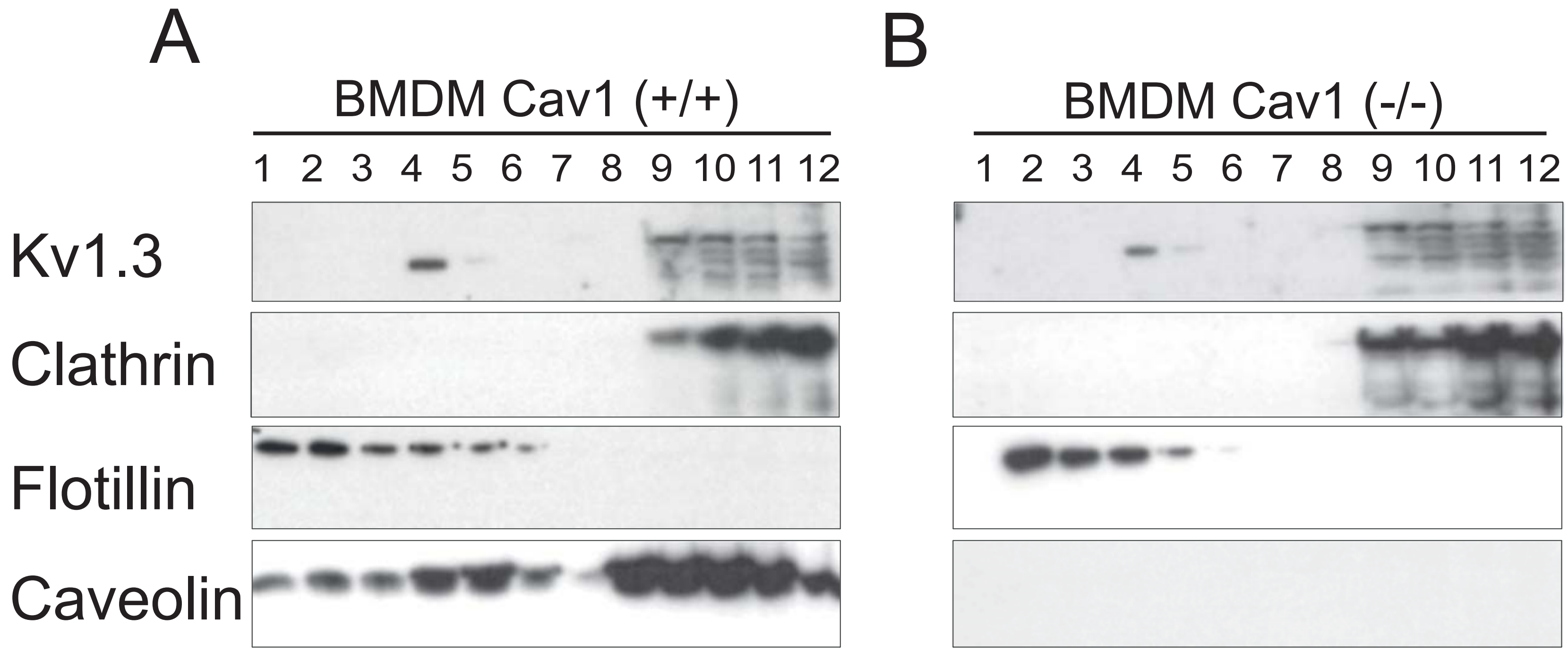
A
BMDM Cav1 (+/+)
1 2 3 4 5 6 7 8 9 10 11 12
Kv1.3
Clathrin
Flotillin
Caveolin
B
BMDM Cav1 (-/-)
1 2 3 4 5 6 7 8 9 10 11 12

Figure S3

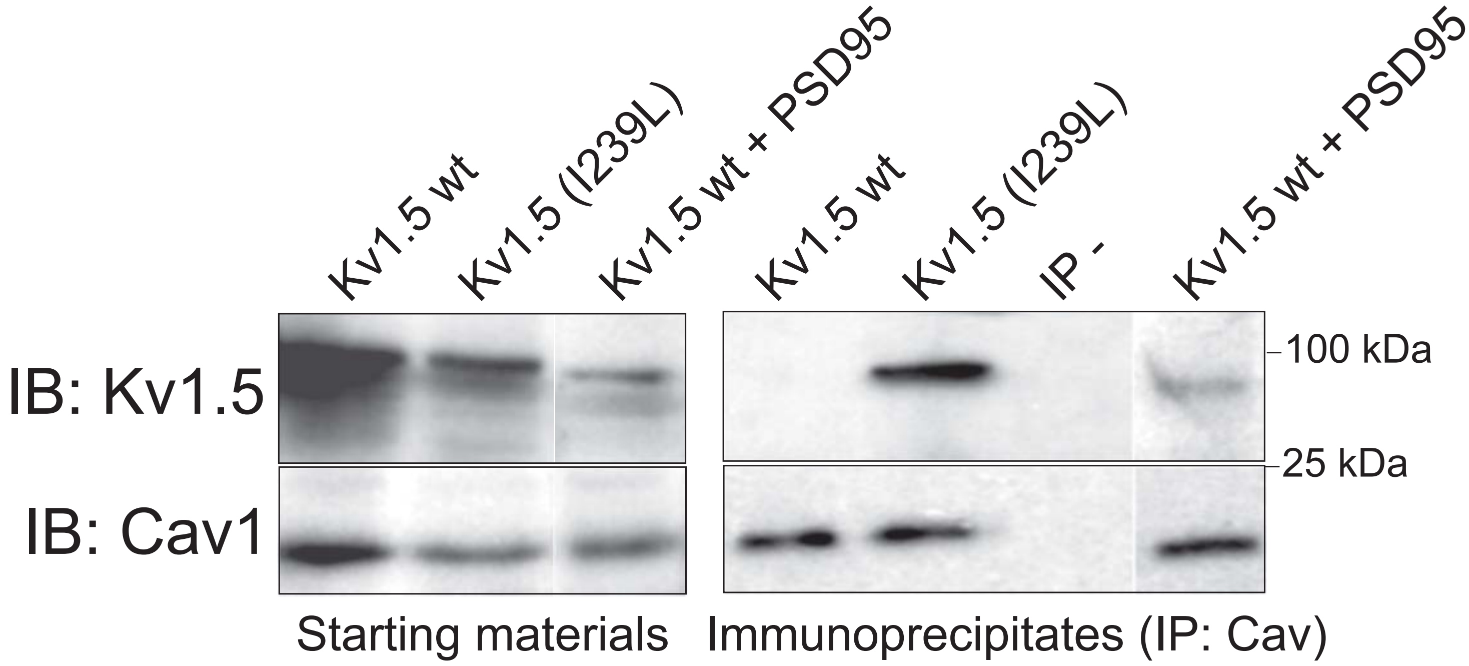
Kv1.5 wt
Kv1.5 (I239L)
Kv1.5 wt + PSD95
Kv1.5 wt
Kv1.5 (I239L)
IP -
Kv1.5 wt + PSD95
IB: Kv1.5
IB: Cav1
100 kDa
25 kDa
Starting materials
Immunoprecipitates (IP: Cav)

Figure S4
